# Microwave treatment regulates the free volume of rice starch

**DOI:** 10.1038/s41598-019-40598-3

**Published:** 2019-03-07

**Authors:** Bowen Yan, Huijie Shen, Daming Fan, Yuan Tao, Yejun Wu, Mingfu Wang, Jianxin Zhao, Hao Zhang

**Affiliations:** 1 State Key Laboratory of Food Science and Technology, Jiangnan University, Wuxi, 214122 China; 2 School of Biological Sciences, The University of Hong Kong, Pokfulam, Hong Kong SAR, Hong Kong, China; 3 National Engineering Research Center for Functional Food, Jiangnan University, Wuxi, 214122 China; 4 School of Food Science and Technology, Jiangnan University, Wuxi, 214122 China; 5 Collaborative Innovation Center of Food Safety and Quality Control in Jiangsu Province, Wuxi, 214122 China

## Abstract

The aim of this work was to investigate the role of microwave parameters and moisture content on the free volume (FV) changes of rice starch by positron annihilation lifetime spectroscopy analysis (PALS) and to explore the potential relationship between the changes of FV and physicochemical properties of rice starch. Microwave heating and water molecules lead to the increasing of FV of starch. However, this result is largely influenced by the plasticization of water molecule. The anti-plasticization caused by water evaporation resulting in a decrease in the size and concentration of FV during microwave heating. Significant decrease (p < 0.05) in the thickness of amorphous region of microwave-heated rice starch was found by small angle X-ray scattering (SAXS), and the glass transition temperature (*Tg*) and gelatinization temperature significantly increase (p < 0.05) after microwave heating. According to correlation analysis, the power intensity and heating time were correlated negatively with the lifetime of o-Ps. In addition, the changes of amorphous region and *Tg* of rice starch were strongly related to FV changes. These results provided a theoretical basis for further research on the directional regulation of FV and improvement the quality of starch-based food by using microwave treatment.

## Introduction

Free volume (FV), the concept of which was first proposed by Fox and Flory with the motivation for illustrating the glass transition process, was defined as the unoccupied volume composed by interspace of molecular chain or imperfections in random arrangement of molecular^1,2^. It has been confirmed that FV is temperature-dependent, and the temperature at which the FV is insufficient for changing molecular conformation, is termed as glass transition temperature (*T*_*g*_)^3^. Therefore, the changes of FV hole will interfere the dynamic and physical properties of polymer. For example, the viscosity of polymer system reduces upon increasing in FV, which is attributed to the enhancement on diffusion ability of the molecular chains^4^.

Starch is a complexly semi-crystalline biopolymer consisting of amorphous amylose and crystalline amylopectin lamellae^5^. The starches with diverse amylose/amylopectin ratios possess the different FV, which will determine the structure, phase transitions and physicochemical properties of starches, and ultimately affect the functional characteristics of starchy food^6^. An earlier study in 1999, Benczedi reported that FV of starch became larger with a higher water concentration^7^, while in other blended starch systems, the role of water was found to be significantly more complex. Hughes indicated that the holes in starch/sucrose blend decreased firstly and then increased with rising moisture content^8^. Therefore, it was speculated that water could influence the starch FV in two ways through the nanoholes occupation and inter-molecular hydrogen bonding formation^9^.

Actually, temperature significantly changes the FV of polymer materials^10^. The main reason for temperature dependence is that segments of the molecular chains are activated to initiates the thermal motion when the thermal energy is sufficient, which determines the size and distribution of the FV in the polymer. Therefore, various heating approaches, heating rate and heating time are bound to change the FV holes. Microwave heating has an extensive application in food processing because of its rapid heating rate and safe handling^11^. In our previous works, we have demonstrated that microwave can change the lamellar architecture of rice starch^12^, which suggested that it might play an important role in regulating the FV of starch. However, there are no available publications regarding the effects of microwave heating on the FV holes of starch although it can manipulate FV of other polymer materials^13^. In addition, it is noteworthy that water is a vital dipole molecule, which had a high response to the applied microwave electromagnetic field^14^. Moisture content, impacting the hydration process of starch, is an important factor in the process of microwave heating starch as we previously reported^15^. However, it is still not clear, microwave heating and water molecule, which plays a more important roles in the FV changes of starch.

Currently, positron annihilation lifetime spectroscopy (PALS) is a highly suitable technique for characterization of FV holes in starch^9,16^, which involves the detection of the lifetimes of positron injected into a polymer. The first objective of this study was to evaluate the effect of moisture content and microwave parameters on the FV changes of rice starch during microwave heating. The second objective was to provide insights into the relationship between the changes of FV and physicochemical properties of rice starch upon the microwave processing.

## Materials and Methods

### Materials

Native rice starch (up to 95% pure) was purchased from BENEO Asia Pacific Pte. Ltd. The amylose and protein contents (dry mass) of the starch were detected according to method reported by Lin *et al*.^17^. The moisture content of rice starch was adjusted to 10%, 15%, 20%, 25%, 30% and 35% and equilibrated for 12 h at ambient temperature.

### Methods

#### Microwave treatment

Batches, one gram, of rice starch were processed at a varieties of power intensity (50, 100, 150, 200, and 250 W/g) by using a Milestone single-mode/multimode microwave synthesizer chamber (Milestone, Sprisole, Italy), which was equipped with an infrared temperature probe to real-time monitor the temperature of samples^18^. After cooling at ambient temperature, parts of samples were screened directly through an 80 mesh sieve for PALS and *Tg* analysis. The others were dried at 30 °C until the moisture content stabilized, and then screening for small angle X-ray scattering (SAXS) and differential scanning calorimeter (DSC) analysis.

#### Measurement of the FV with PALS

The lifetime measurements were made with a standard fast-fast coincidence system, and the positron source ^22^Na was deposited in a BaF_2_ probe^9^. Eight grams of starch samples were sealed with Mylar film (7 mm thick) to prevent water evaporation during measurement; the source was placed in the center of the sample to form a sandwich structure. The time resolution [full width at half maximum (FWHM)] of prompt spectra was 0.23 ns and the time spectrum was measured until ~10^6^ total counts were accumulated in the peak channel. The lifetime τ_3_ and the intensity I_3_ of o-Ps represent the FV hole size and the number of FV cavities per unit volume of starch, respectively, which were analyzed by using the spectral software PATFIT (discrete spectrum) and LT (continuous spectrum).

According to the Tao-Eldrup formula, the relationship between the value τ_3_ and the FV of the hole radius is calculated^19,20^.

1 $${{\rm{\tau }}}_{3}({\rm{ns}})={\{2[1-\frac{{\rm{R}}}{{\sum }^{}}+\sin (\frac{2{\rm{\pi }}{\rm{R}}}{{\sum }^{}})/(2{\rm{\pi }})]\}}^{-1}$$

Σ = R + ΔR and ΔR is 0.166 nm, which is an empirical parameter; that is, the thickness of the uniform electron layer in the spherical potential well is assumed, and o-Ps is trapped solely in the electron layer.

2 $${{\rm{V}}}_{{\rm{f}}}=4{{\rm{\pi }}{\rm{R}}}^{3}/3$$

The FV radius R is calculated with formula (1), and the FV size is calculated with formula (2).

3 $${{\rm{f}}}_{{\rm{v}}}={\rm{C}}\times {{\rm{I}}}_{3}\times {{\rm{V}}}_{{\rm{f}}}$$

The volume fraction of the FV, that is, its percentage of the total volume of the polymer, is expressed by f_v_, which can be calculated by formula (3), where C is a constant 0.018 nm^−3^ ^21^.

#### Small angle X-ray scattering

For SAXS analysis, the moisture content of rice starch was adjusted to 50% in order to remove the water in the upper layer of starch. The lamellar structure of rice starch was performed on a SAXS nanostructure analyzer (Anton Paar, Austria), with an incident X-ray beam (λ_CuKα_ = 1.54 Å) monitored by a photomultiplier. The rotating anode device was operated at 50 kV and 0.6 mA, and the exposure time was 15 min. The scattering vector in the range of 0.07 to 2.3 nm^−1^ was detected (${\rm{q}}=\frac{4\pi \,\sin \,\theta }{\lambda }$) with λ as the wavelength and θ as the scattering angle). Transmission, dark current and aluminum foil corrections were performed on the 2D image first. Allowing for further SAXS data processing, contributions from background density fluctuations were removed. Experimental SAXS curves were then desmeared with the SAXSQuant software (Anton Paar, Austria) for collimation distortions^22^. Based on the pseudo-two-phase model for lamellar semi-crystalline polymers, the morphological parameters of starch was obtained by the calculation of linear correlation function γ(x). The formula of the one-dimensional correlation function is as follows, where x represents the actual distance, q is the scattering vector, and I is the scattering intensity.

4 $${\rm{\gamma }}({\rm{x}})=\frac{{\int }_{0}^{\infty }{\rm{I}}({\rm{q}}){{\rm{q}}}^{2}\,\cos ({\rm{qx}}){\rm{dq}}}{{\int }_{0}^{\infty }{\rm{I}}({\rm{q}}){{\rm{q}}}^{2}{\rm{dq}}}\,$$

The information related to the Small angle X-ray scattering (SAXS) spectra of the starch samples was analyzed with a one-dimensional function, and the related parameters were calculated by referring to the methods of Zhu *et al*.^23^ dc is the crystalline thickness of the lamellar structure; the value corresponds to the intersection of the first minimal (peak valley) horizontal line and the oblique line on the Z coordinate. da is the average thickness of the amorphous region (da = L − dc). L is the average period of the layered structure (long period); the value corresponds to the first maximum (peak) of γ(x) on the Z coordinate. Φ represents the volume fraction of the crystalline zone in the long period, that is, the ratio of dc to L. The graphical representation of the mentioned parameters was shown in Figure S1.

#### Glass transition temperature

*Tg* of the native starch-water system was carried by using DSC (PE-8500, power-compensated; PerkinElmer, United States). 5 ± 0.2 mg of starch samples was sealed in an aluminum pan and heated at a rate of 10 °C/min from 10 °C to 130 °C, then samples were cooled at 20 °C/min to 10 °C, and heated at 5 °C/min to 130 °C according to two heating scan method^24^. The *Tg* values were estimated from the midpoint of the heat capacity change observed during the second heating scan.

#### Gelatinization properties

The gelatinization properties of starch were performed as described to evaluate molecular activity by using a DSC (PE-8500, power-compensated; PerkinElmer, United States). The moisture content of sample was measured before DSC analysis, and appropriate amount of rice starch was mixed with deionized water to ensure a weight ratio of starch and water was 1:2. 8–10 mg samples were placed in an aluminum pan and heated at 30 °C for 5 min, followed by heating from 30 °C to 90 °C at a scanning rate of 10 °C/min^17^. The parameters of T_o_ (onset temperature), T_p_ (peak temperature), T_c_ (conclusion temperature), and ΔH (enthalpy) were obtained based on the DSC analysis.

#### Statistical analysis

OriginPro 8 SR1 (OriginLab Corporation, Northampton, MA, USA) and Microsoft Office Excel® 2013 was used to conduct the graphical analysis and statistical calculations. Values were expressed as mean values ± standard deviations. Significant differences among values were calculated based on Tukey’s procedure at p < 0.05, using the software SPSS statistics (version 17). All the measurements were repeated at least three times for each sample.

## Results and Discussion

### Effect of moisture content on the FV of rice starch

**Figure 1:**
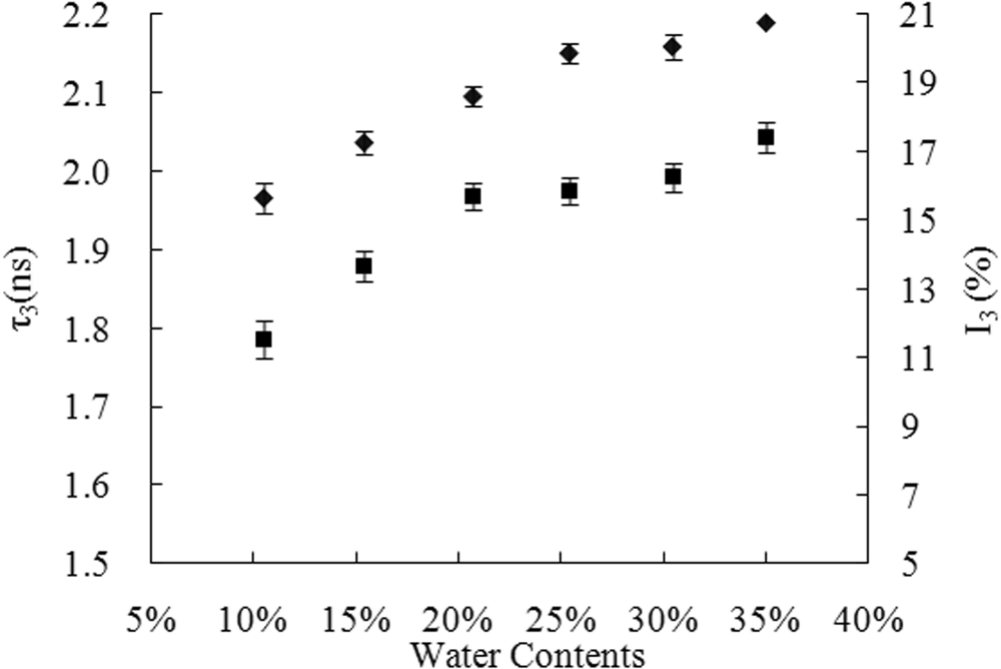
Effect of moisture content on the free volume of rice starch. The o-Ps lifetime, τ_3_ (ns), and intensity, I_3_ (%), of rice starch with various moisture contents. (■ represents o-Ps lifetime, τ_3_ (ns), ◆ represents o-Ps intensity, I_3_ (%)).

**Figure 2:**
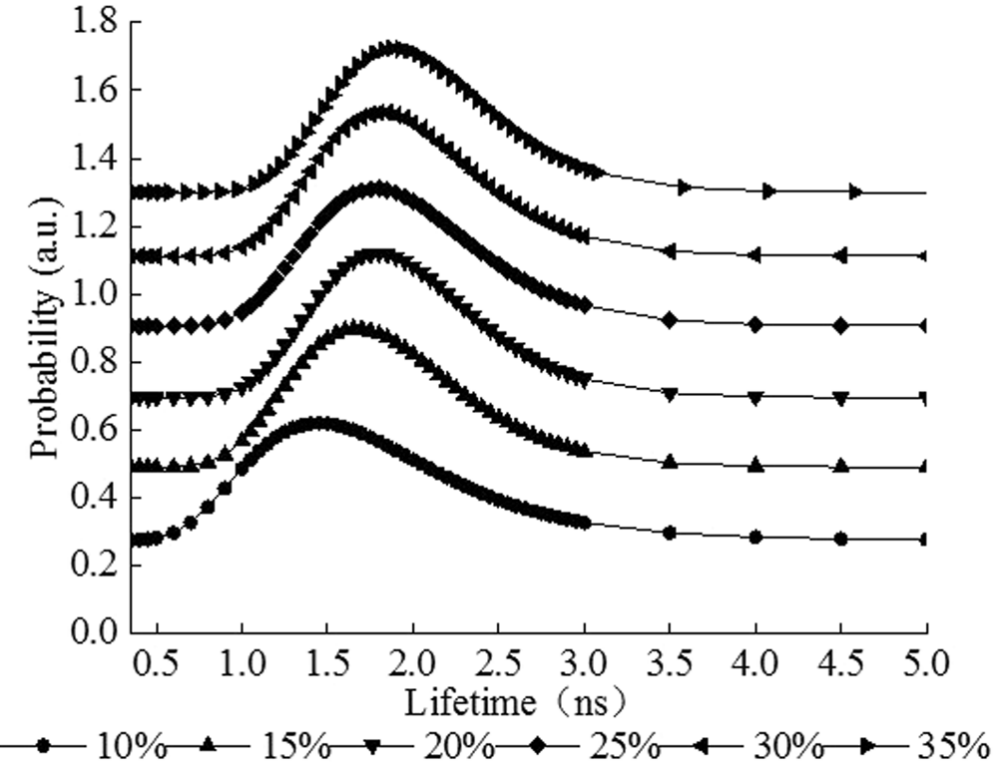
Continuous o-Ps lifetime distribution of rice starch with different moisture contents, i.e., 10%, 15%, 20%, 25%, 30%, and 35%.

The FV of rice starch with different moisture content was evaluated by PLAS analysis (Fig. 1). The increasing of moisture content resulted in higher τ_3_ and I_3_ values of starch samples, which indicated that the FV in the starch was expanded by water adsorption. Water molecules regulate the FV of starch through occupying the free spaces and changing the hydrogen bond interactions in the biopolymer^9^. The I_3_ value of pure water is 26.9%, indicating the generation probability of positronium in the pure water is higher than that in the plasticized starch^25^. The I_3_ values of samples were less than those in the pure water and increased with increasing moisture content, which indicated that the FV of starch was not filled with water molecules. The I_3_ value may be the result of the average of o-Ps intensity between the small molecules and starch polymer. To further evaluate the effect of moisture content on FV in the rice starch, the size distribution of the FV in starch systems with various moisture contents was analyzed from the continuous distribution spectrum (Fig. 2). The lifetime of starch presented an asymmetric distribution in the range of 0.5 to 3 ns and a tail was found at the peak value of lifetime, which were consistent with the previous report^9^. The peak of lifetime distribution shifted to the right and was reduced in width with increase of moisture content, which indicated that the water molecules resulted in the FV of rice starch increased. The combination of discrete spectrum and continuous spectrum analysis for the lifetime of starch clearly illustrated the effect of moisture content on the FV of rice starch, and the FV changes of samples become gently with the moisture content in the range of 25% to 35%. To minimize the influence of moisture evaporation on the changes of FV, the starch with specific moisture contents (25%, 30%, and 35%) were selected for the following studies.

### Effect of microwave parameters on the FV of rice starch

**Figure 3:**
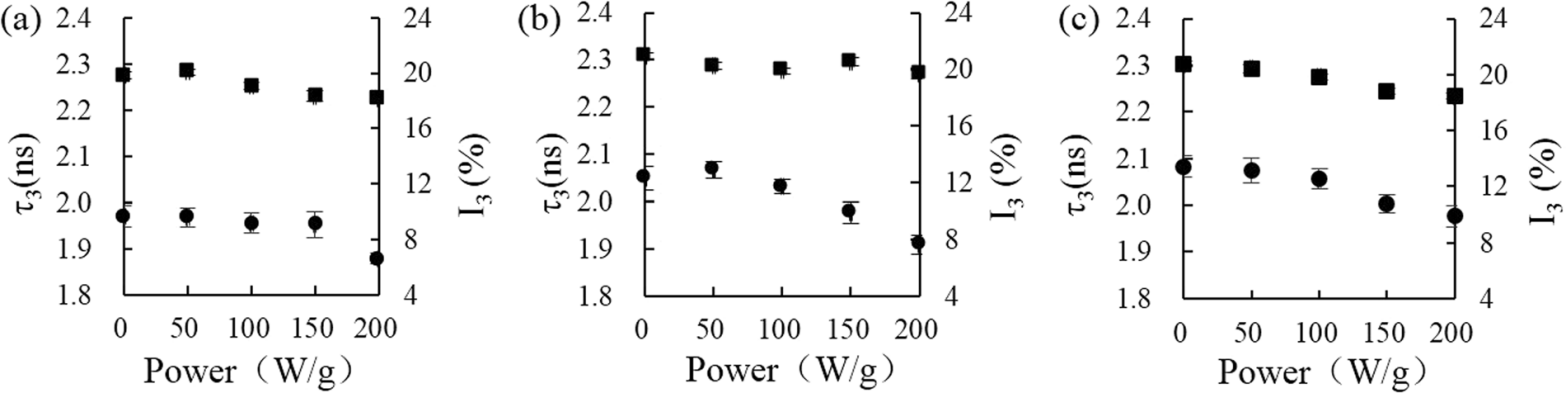
Effect of microwave power on the free volume of rice starch. The o-Ps lifetime, τ_3_ (ns), and intensity, I_3_ (%), of rice starch, with moisture contents of 25% (**a**), 30% (**b**), and 35% (**c**), respectively, after heating for 5 min at different microwave power from 0 to 200 W/g. (■ represents o-Ps intensity, I_3_ (%), ● represents o-Ps lifetime, τ_3_ (ns)).

**Figure 4:**
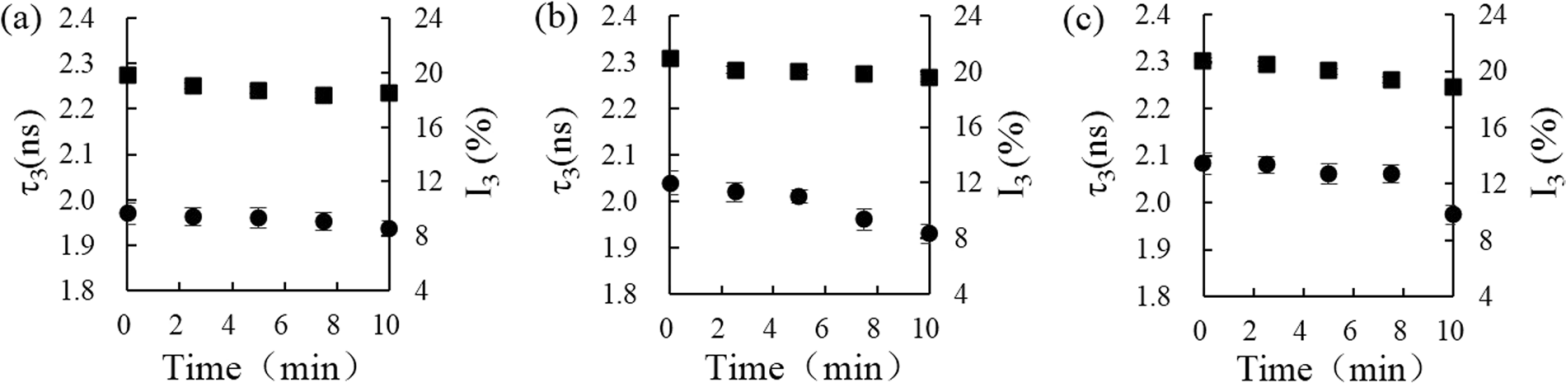
Effect of microwave time on the free volume of rice starch. The o-Ps lifetime, τ_3_ (ns), and intensity, I_3_ (%) of rice starch, with moisture contents of 25% (**a**), 30% (**b**), and 35% (**c**), respectively, after microwave heating at 100 W/g for 0 to 10 min. The average o-Ps lifetime, τ_3_ (ns), and intensity, I_3_ (%), of the samples with three moisture contents decreased after heating for various times in microwave fields. (■ represents o-Ps intensity, I_3_ (%), ● represents o-Ps lifetime, τ_3_ (ns)).

The FV changes of rice starch were evaluated at varieties of power intensity (50, 100, 150, 200, and 250 W/g) and in a range of heating time (0 to 10 min) by PLAS analysis. The lifetime and intensity values of o-Ps appeared to a downward trend as the power intensity increased (Fig. 3), suggesting that molecular interactions in the rice starch were improved, such as the cross-linking through hydrogen bond formation. Raj and Ranganathaiah also found the similar results, namely the behavior of positron parameters (τ_3_ and I_3_) decreased in the microwave-irradiated samples^26^. The FV of rice starch at varieties of power intensity were calculated by the Tao-Eldrup formula (Table S1)^19,20^. We observed that the FV of sample, with a moisture content of 25%, decreased significantly by microwave heating at 200 W/g, but for the samples with the moisture content of 30% and 35%, the FV changes varied significantly at the power intensity of 100 W/g. As the previous study reported that water molecules dominated the electromagnetic properties of rice starch, low moisture content of starch resulted in not very effective microwave absorption^27^. The similar FV changes of rice starch were also observed by microwave heating in a range of heating time (0 to 10 min) (Fig. 4). The FV of samples with specific moisture contents (25%, 30%, and 35%) decreased from 94.71 to 91.64 Å^3^, 102.17 to 91.01 Å^3^, and 105.38 to 95.24 Å^3^, respectively (Table S2). Therefore, the increasing of power intensity and heating time of microwave treatment reduced the size and concentration of FV of rice starch. However, the previous results proved the plasticization of water molecule resulted in the FV of rice starch increasing^9^, thus the evaporation of water may be the main reason for the FV decreasing of rice starch during heating by microwave.

**Figure 5:**
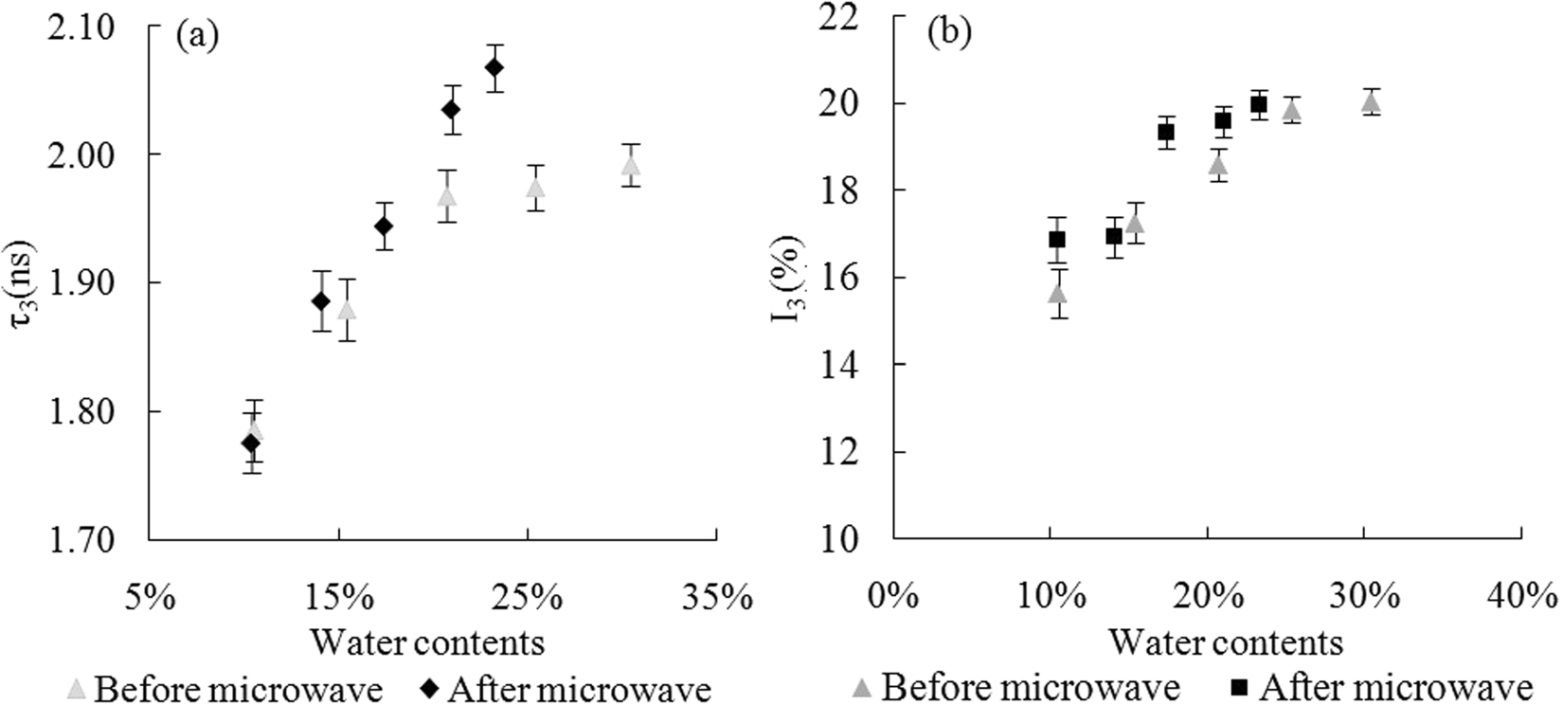
The o-Ps lifetime, τ_3_ (ns), (**a**) and intensity, I_3_ (%), (**b**) of rice starch with different moisture contents from ~10% to ~30% before and after microwave radiation at 100 W/g for 5 min. The moisture contents of rice starch before microwave heating were 10.50%, 15.40%, 20.70%, 25.38%, and 30.42%. The corresponding moisture contents of rice starch after heating at 100 W/g for 5 min were 10.39%, 14.09%, 17.41%, 20.97%, and 23.26%, respectively.

To further verify whether the FV changes of rice starch were caused by the evaporation of water after microwave heating, the FV of samples with various of moisture contents were analyzed. As the results shown in Fig. 5, microwave heating resulted in higher lifetime and intensity values of samples as compared to the unheated rice starch with specific moisture content, which indicated the FV increased by microwave heating, especially in a higher moisture content situation (moisture content >20%). As we known, more water molecules will be available for microwave absorption leading to maximum interaction by comparison of samples with less moisture content^28^. In addition, the effective of microwave absorption resulted in the temperature of samples raised rapidly, which had a positive effect on the FV^9^. However, our previous study observed that the changes of FV size and concentration were different by comparing microwave and conventional heating methods^29^. Therefore, it could be concluded that the plasticization of water molecules play a synergetic effect in heating process and the microwave heating resulted in the FV changes of rice starch, but the anti-plasticization caused by the evaporation of water is the main reason for the reduction of FV in the samples during microwave heating.

### Changes of lamellae structure in rice starch by microwave heating

The effect of microwave heating on the lamellae structure of rice starch was investigated by SAXS. There was no significant difference in the scattering peak position of the sample with the increasing of power intensity and heating time, which indicated that the thickness of lamellae structure of rice starch was little changed by microwave heating. One dimensional function (Fig. S1) was used to obtain the morphological parameters of starch, in the case of pseudo-two-phase model (sharp boundaries at the crystal/amorphous interface with an electron density gradient transition layer) for lamellar semi-crystalline polymers like starch^30^. As shown in Tables 1 and 2, microwave power intensity had no significant effect on the long period of lamellar structure of starch with the values range of 8.731–8.769 nm. The volume fractions of the crystalline phase in the long period was about 40%, whereas the crystallinity of starch was about 22% in the previous study^31^, which implied that the structure of starch was matching with the three-phase model. This model consists of crystalline region, moving amorphous region, and amorphous zone with a semi-crystalline lamellar structure, which is consistent with previous reports^32^. However, compared to the native starch, the average thickness of amorphous region of microwave-heated rice starch had a tendency to decrease, which was possible caused by the compression of the amorphous region after microwave heating. Therefore, it is speculated that there is a correlation between FV and amorphous region of starch lamellar structure.

**Table 1:** Structural features of the lamellae of rice starch (water content = 30%) after microwave heating for 5 min at different powers, i.e., 0, 50, 100, 150, and 200 W/g.

Sample	Native starch	50 W/g	100 W/g	150 W/g	200 W/g
L (nm)	8.750 ± 0.019^a^	8.750 ± 0.014^a^	8.750 ± 0.017^a^	8.750 ± 0.013^a^	8.750 ± 0.015^a^
dc (nm)	3.536 ± 0.022^a^	3.605 ± 0.012^b^	3.617 ± 0.022^b^	3.659 ± 0.014^c^	3.609 ± 0.021^b^
da (nm)	5.214 ± 0.003^d^	5.145 ± 0.002^c^	5.134 ± 0.006^b^	5.091 ± 0.001^a^	5.141 ± 0.006^bc^
Φ	0.404 ± 0.002^a^	0.412 ± 0.001^b^	0.413 ± 0.002^b^	0.418 ± 0.001^c^	0.412 ± 0.002^b^

The different superscript letter within a column indicates significant difference(p < 0.05).

L: long period, i.e. the lamellar repeat distance.

da: the thickness of amorphous layers.

dc: the thickness of crystalline layers.

Φ: volume fractions of the crystalline phase in the semi-crystalline stacks of starch.

**Table 2:** Structural features of the lamellae of rice starch (water content = 30%) after microwave heating at 100 W/g for different times, i.e., 0, 2.5, 5, 7.5, and 10 min.

Sample	Native starch	2.5 min	5 min	7.5 min	10 min
L (nm)	8.750 ± 0.019^a^	8.750 ± 0.017^a^	8.750 ± 0.017^a^	8.750 ± 0.016^a^	8.750 ± 0.015^a^
dc (nm)	3.536 ± 0.022^a^	3.665 ± 0.018^c^	3.617 ± 0.022^b^	3.710 ± 0.021^d^	3.612 ± 0.013^b^
da (nm)	5.214 ± 0.003^c^	5.085 ± 0.001^ab^	5.134 ± 0.006^b^	5.040 ± 0.005^a^	5.030 ± 0.062^b^
Φ	0.404 ± 0.002^a^	0.419 ± 0.001^bc^	0.413 ± 0.002^b^	0.424 ± 0.002^c^	0.425 ± 0.007^b^

The different superscript letter within a column indicates significant difference(p < 0.05).

L: long period, i.e. the lamellar repeat distance.

d_a_: the thickness of amorphous layers.

d_c_: the thickness of crystalline layers.

Φ: volume fractions of the crystalline phase in the semi-crystalline stacks of starch.

### Changes of Tg in rice starch by microwave heating

**Figure 6:**
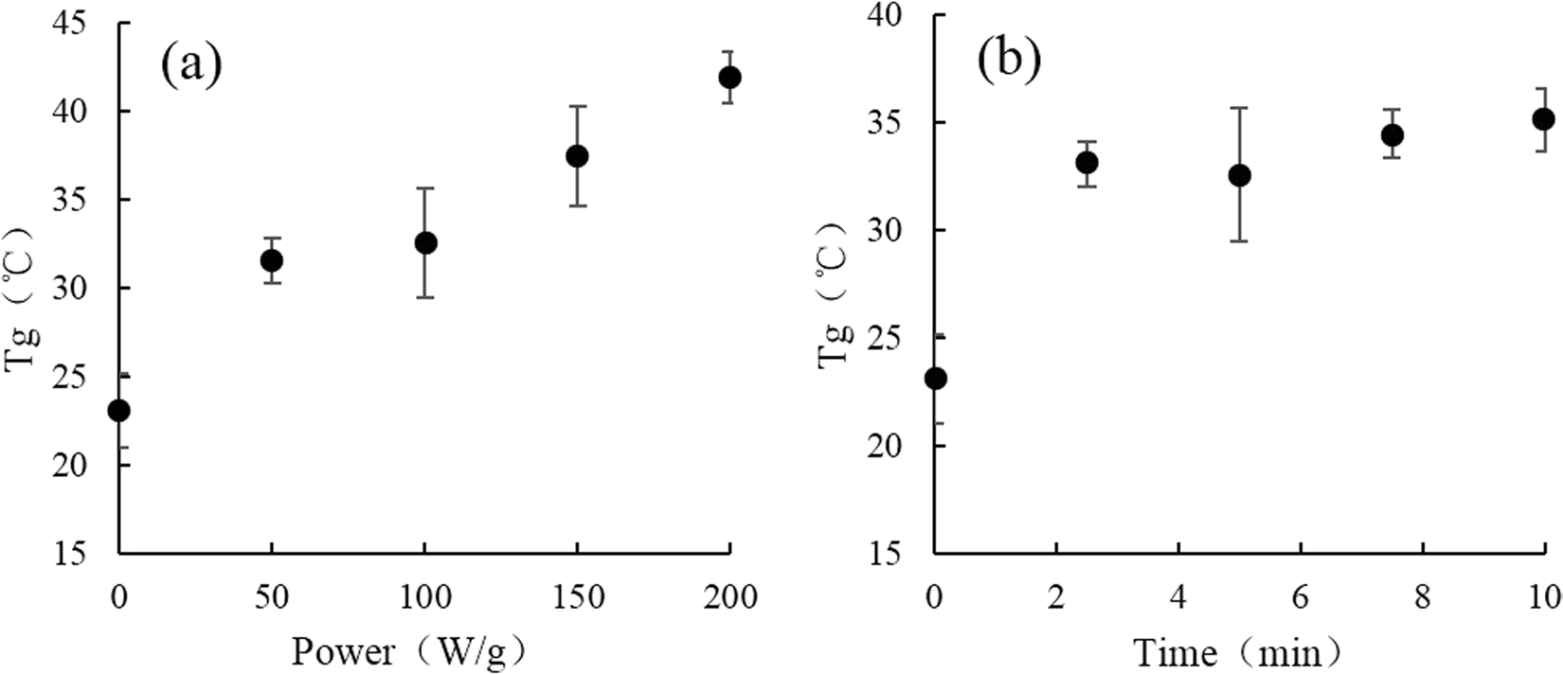
Glass temperature (*Tg*) of rice starch after microwave heating for 5 min at 0 to 200 W/g (**a**), and for 0 to 10 min at 100 W/g (**b**).

The *Tg*, as an important characteristic of amorphous polymers, contributes to the physical properties of food^33^. As shown in Fig. 6a, *Tg* increased with the microwave power intensity. This result is consistant with previously reported by Zhao *et al*., which the power intensity affected the water distribution and *Tg* was negatively correlated with the areas of cytoplasmic bulk water^34^. The fluctuation was observed during microwave heating in a range of time (Fig. 6b), but the general trend of *Tg* was increased. As the previously reported that the changes of the *Tg* had a strong association with FV^35^, and the variation of *Tg* was corresponding to FV changes of rice starch. In addition, configurational rearrangements of starch chain backbones will occur extremely slowly at this critical temperature^36^. Therefore, the changes in *Tg* may be attributed to the decrease of FV and molecular mobility of rice starch.

### Changes of gelatinization properties in rice starch by microwave heating

After microwave heating, the gelatinization properties of rice starch were investigated by DSC analysis. As the results shown in Tables 3 and 4, microwave heating led to a significant increasing in the gelatinization temperature and ΔH of starch samples, as compared to the native starch. Starch with heat-moisture treatment used to exhibit an upward shift in gelatinization temperature since the increased associations among starch polymer chains^37^. Based on the microwave heating, more polar groups of rice starch will be available for microwave absorption leading to maximum interaction to rearrange the crystallization and amorphous area of starch granules to form a more orderly crystal matrix^38^. The rearrangement resulted in the increasing of crystalline area and the amorphous region reduction of rice starch, and the changes of structure possibly resulted in the decrease of the FV and molecular mobility^5^. These results seem that the increased in *Tg* and gelatinization enthalpy might be related to the changes of the FV in rice starch. In addition, excessive power intensity may induce partial structural degradation of starch, which resulted in the reduction of gelatinization enthalpy^39^.

**Table 3:** Onset temperature (T_o_), peak temperature (T_p_), conclusion temperature (T_c_), and enthalpy change (ΔH) of rice starch (water content = 30%) after microwave heating for 5 min at different power, i.e., 0, 50, 100, and 200 W/g.

Samples	T_o_ (°C)	T_p_ (°C)	T_c_ (°C)	ΔH (J/g)
Native starch	71.06 ± 0.10^a^	74.87 ± 0.12^a^	79.25 ± 0.24^a^	11.14 ± 0.14^a^
50 W/g	72.25 ± 0.15^b^	76.00 ± 0.19^b^	80.64 ± 0.18^b^	11.98 ± 0.03^ab^
100 W/g	72.51 ± 0.20^b^	75.98 ± 0.20^b^	80.52 ± 0.31^b^	12.72 ± 0.53^b^
150 W/g	72.14 ± 0.07^b^	75.74 ± 0.03^b^	80.07 ± 0.16^a^	11.70 ± 0.47^ab^
200 W/g	72.48 ± 0.04^b^	76.12 ± 0.01^b^	81.11 ± 0.08^b^	10.81 ± 0.23^a^

The different superscript letters within a column indicate significant difference (p < 0.05).

**Table 4:** Onset temperature (T_o_), peak temperature (T_p_), conclusion temperature (T_c_), and enthalpy change (ΔH) of rice starch (water content = 30%) after microwave heating for 5 min at 100 W/g for different time, i.e., 0, 2.5, 5, 7.5, and 10 min.

Samples	T_o_ (°C)	T_p_ (°C)	T_c_ (°C)	ΔH (J/g)
Native starch	71.06 ± 0.10^a^	74.87 ± 0.12^a^	79.25 ± 0.24^a^	11.14 ± 0.14^a^
2.5 min	72.49 ± 0.02^b^	76.11 ± 0.01^b^	80.61 ± 0.08^b^	12.01 ± 0.03^ab^
5 min	72.51 ± 0.20^b^	75.98 ± 0.20^b^	80.52 ± 0.31^b^	12.72 ± 0.53^b^
7.5 min	72.50 ± 0.09^b^	76.01 ± 0.10^b^	80.53 ± 0.09^b^	12.29 ± 0.57^ab^
10 min	72.47 ± 0.09^b^	75.98 ± 0.07^b^	80.41 ± 0.02^b^	12.16 ± 0.19^ab^

The different superscript letters within a column indicate significant difference (p < 0.05).

### Correlation analysis between FV and physical properties of rice starch by microwave heating

The results of the correlation analysis (Tables 5 and 6) showed that power intensity and heating time were correlated negatively with the lifetime of o-Ps, which indicated that microwave heating regulated the FV hole size of rice starch. This effect was largely influenced by the heating time as compared to power intensity, which means that temperature changes and anti-plasticization caused by water evaporation during microwave heating are the main reasons for the changing of FV in rice starch. This similar result was also consistent with other studies on the maltodextrin, which reported that the average volume of the holes between the polymer chains increase with the moisture content of the matrix^40^. For the correlation between FV and specific physical properties of rice starch based on the results of samples with different heating time, da had a significant positive correlation with τ_3_ and I_3_, which suggested that amorphous region had a strong correlation with the size and concentration of FV. In addition, the shrink of FV in polymers will have an impact on the chain segment movement, resulting rise in viscosity and *Tg*^33^, thus significant negative correlation was observed between *Tg* and the FV concentration of rice starch.

**Table 5:** Correlation analysis of free volume, structure, and properties of rice starch with the changes of microwave power.

Factor	P	τ_3_	I_3_	dc	da	*T*_*g*_	ΔH
P	1						
τ_3_	−0.913*	1					
I_3_	−0.716	0.550	1				
dc	0.714	−0.413	−0.418	1			
da	−0.715	0.415	0.414	−1**	1		
*Tg*	0.976**	−0.842	−0.747	0.763	−0.765	1	
ΔH	−0.200	0.547	−0.120	0.359	−0.352	−0.153	1

*Significant correlation at 0.05 level (bilateral); **Significant correlation at 0.01 level (bilateral).

**Table 6:** Correlation analysis of free volume, structure, and properties of rice starch with the changes of microwave time.

Factor	t	τ_3_	I_3_	dc	da	*T*_*g*_	ΔH
t	1						
τ_3_	−0.982**	1					
I_3_	−0.935*	0.894*	1				
dc	0.479	−0.483	−0.628	1			
da	−0.864	0.892*	0.917*	−0.773	1		
*Tg*	0.821	−0.783	−0.962**	0.788	−0.930*	1	
ΔH	0.632	−0.498	−0.793	0.614	−0.584	0.826	1

*Significant correlation at 0.05 level (bilateral); **Significant correlation at 0.01 level (bilateral).

## Conclusions

This study provides novel and deeper insights on the FV changes of rice starch during microwave heating. More importantly, we reveal that the microwave heating and the plasticization of water molecules play a synergetic effect on the FV changes of rice starch, and the physical properties of rice starch were also strongly related to FV changes in the samples. These results may facilitate optimization of starch-based microwave food process to achieve better quality features. The detailed regulation between the FV changes involved in microwave parameters and the quality of products requires further investigation with improved strategies.

## Supplementary information

Supporting Information

## Data Availability

All data generated or analyzed during this study are included in this published article.
