# Supplementary material for: Microwave treatment regulates the free volume of rice starch
Source: Sci Rep. 2019 Mar 7;9:3876. doi: 10.1038/s41598-019-40598-3 (PMC6405908; doi:10.1038/s41598-019-40598-3)
Supplement: Supplementary file 1 — Supporting Information [file 41598_2019_40598_MOESM1_ESM.docx]

**Supporting Information**

**for**

**Microwave treatment regulates the free volume of rice starch**

Bowen Yan^1,3,4^, Huijie Shen^1,3,4^, Daming Fan^1,2,3,4,5*^, Yuan Tao ^1,3,4^, Yejun Wu ^1,3,4^, Mingfu Wang^2*^, Jianxin Zhao^1,3,4,5^, Hao Zhang^1,3,4,5^

1.State Key Laboratory of Food Science and Technology, Jiangnan University, Wuxi 214122, China;

2.School of Biological Sciences, The University of Hong Kong, Pokfulam, Hong Kong SAR, China;

3.National Engineering Research Center for Functional Food, Jiangnan University, Wuxi 214122, China;

4.School of Food Science and Technology, Jiangnan University, Wuxi 214122, China;

5.Collaborative Innovation Center of Food Safety and Quality Control in Jiangsu Province, Wuxi 214122, China.

*Co-corresponding authors:

Prof. Daming Fan Tel.: +86 0510 85884620; fax: +86 0510 85326696;

E-mail address: [fandm@jiangnan.edu.cn](mailto:fandm@jiangnan.edu.cn)

Dr. Mingfu Wang Fax: +852 22990348; E-mail address: [mfwang@hku.hk](mailto:mfwang@hku.hk)

Table S1. Changes in free volume size (V_f_) and fraction (f_v_, the concentration of free volume in total volume of starch) of rice starch with different moisture contents after microwave heating for 5 min at different power intensities, i.e., 0, 50, 100, 150, and 200 W/g.

| Moisture Content (%) | Power (W/g) | V_f_ (Å3) | f_v_=C*Ι_3_*V_f_ (%) |
| --- | --- | --- | --- |
| 25 | 0 | 94.72±2.24 ^a^ | 3.39±0.13 ^a^ |
|  | 50 | 94.44±1.96 ^a^ | 3.41±0.11 ^a^ |
|  | 100 | 93.32±2.04 ^a^ | 3.20±0.11 ^ab^ |
|  | 150 | 93.04±2.69 ^a^ | 3.07±0.15 ^b^ |
|  | 200 | 86.24±1.09 ^b^ | 2.81±0.06 ^c^ |
| 30 | 0 | 102.26±2.38 ^ab^ | 3.85±0.14 ^a^ |
|  | 50 | 104.16±1.72 ^a^ | 3.80±0.10 ^ab^ |
|  | 100 | 100.35±1.43 ^b^ | 3.59±0.09 ^bc^ |
|  | 150 | 95.67±2.15 ^c^ | 3.53±0.13 ^c^ |
|  | 200 | 89.14±1.89 ^d^ | 3.15±0.11 ^d^ |
| 35 | 0 | 105.41±2.21 ^a^ | 3.93±0.13 ^a^ |
|  | 50 | 104.55±2.49 ^a^ | 3.84±0.14 ^ab^ |
|  | 100 | 102.81±2.12 ^a^ | 3.68±0.12 ^b^ |
|  | 150 | 97.81±1.69 ^b^ | 3.31±0.09 ^c^ |
|  | 200 | 95.37±2.05 ^b^ | 3.17±0.11 ^c^ |

The different superscript letter within a column indicates significant difference(p<0.05).

Table S2. Changes in free volume size (V_f_) and fraction (f_v_, the concentration of free volume in total volume of starch) of rice starch with different moisture contents after microwave heating at 100 W/g for different times, i.e., 0, 2.5, 5, 7.5, and 10 min.

| Moisture Content (%) | Time (min) | V_f_ (Å3) | f_v_=C*Ι_3_*V_f_ (%) |
| --- | --- | --- | --- |
| 25 | 0 | 94.72±2.24 ^a^ | 3.39±0.13 ^a^ |
|  | 2.5 | 94.46±1.77 ^a^ | 3.24±0.10 ^ab^ |
|  | 5 | 93.79±2.05 ^a^ | 3.17±0.11 ^b^ |
|  | 7.5 | 93.04±1.86 ^a^ | 3.07±0.10 ^b^ |
|  | 10 | 91.65±1.39 ^a^ | 3.05±0.08 ^b^ |
| 30 | 0 | 101.31±2.36 ^a^ | 3.81±0.14 ^a^ |
|  | 2.5 | 99.41±1.97 ^a^ | 3.60±0.12 ^ab^ |
|  | 5 | 98.46±1.42 ^a^ | 3.54±0.09 ^bc^ |
|  | 7.5 | 93.81±2.21 ^b^ | 3.35±0.12 ^cd^ |
|  | 10 | 91.02±1.92 ^b^ | 3.20±0.11 ^d^ |
| 35 | 0 | 105.39±2.23 ^a^ | 3.93±0.13 ^a^ |
|  | 2.5 | 105.11±1.64 ^a^ | 3.88±0.10 ^ab^ |
|  | 5 | 103.29±2.10 ^a^ | 3.72±0.12 ^bc^ |
|  | 7.5 | 103.30±1.81 ^a^ | 3.60±0.10 ^c^ |
|  | 10 | 95.19±1.86 ^b^ | 3.23±0.10 ^d^ |

The different superscript letter within a column indicates significant difference(p<0.05).


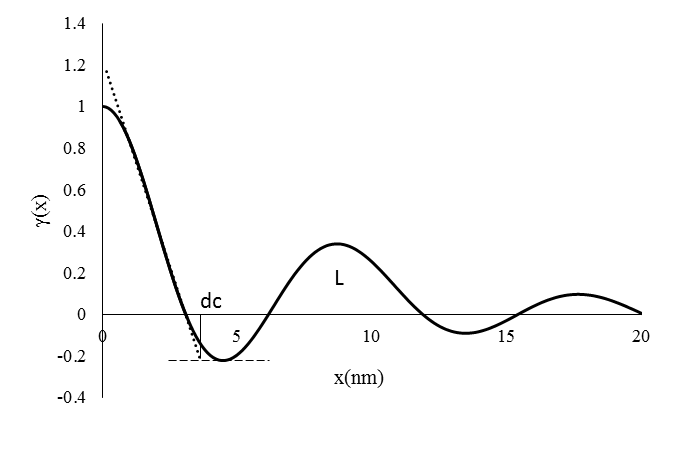


Figure S1. Graphical representation of the lamellar architecture parameters of rice starch from the γ curve
